# Application of two job indices for general occupational demands in a pooled analysis of case–control studies on lung cancer

**DOI:** 10.5271/sjweh.3967

**Published:** 2021-08-31

**Authors:** Jan Hovanec, Jack Siemiatycki, David I Conway, Ann Olsson, Pascal Guenel, Danièle Luce, Karl-Heinz Jöckel, Hermann Pohlabeln, Wolfgang Ahrens, Stefan Karrasch, Heinz-Erich Wichmann, Per Gustavsson, Dario Consonni, Franco Merletti, Lorenzo Richiardi, Lorenzo Simonato, Cristina Fortes, Marie-Élise Parent, John R McLaughlin, Paul Demers, Maria Teresa Landi, Neil Caporaso, Guillermo Fernández-Tardón, David Zaridze, Beata Świątkowska, Tamas Pándics, Jolanta Lissowska, Eleonora Fabianova, John K Field, Dana Mates, Vladimir Bencko, Lenka Foretova, Vladimir Janout, Hans Kromhout, Roel Vermeulen, Paolo Boffetta, Kurt Straif, Joachim Schüz, Swaantje Casjens, Beate Pesch, Thomas Brüning, Thomas Behrens

**Affiliations:** Institute for Prevention and Occupational Medicine of the German Social Accident Insurance (IPA), Institute of the Ruhr University Bochum, Bochum, Germany; University of Montreal, Hospital Research Center (CRCHUM) and School of Public Health, Montreal, Canada; Dental School, College of Medicine Veterinary and Life Sciences, University of Glasgow, Glasgow, UK; International Agency for Research on Cancer (IARC/WHO), Lyon, France; Center for Research in Epidemiology and Population Health (CESP), Exposome and Heredity team, Inserm U1018, University Paris-Saclay, Villejuif, France; Univ Rennes, Inserm, EHESP, Irset (Institut de recherche en santé, environnement et travail) - UMR_S 1085, Pointe-à-Pitre, France; Institute for Medical Informatics, Biometry and Epidemiology, University Hospital Essen, Essen, Germany; Leibniz-Institute for Prevention Research and Epidemiology - BIPS, Bremen, Germany; University Bremen, Faculty 3 - Mathematics and Computer Science, Bremen, Germany; Institute and Clinic for Occupational, Social and Environmental Medicine, University Hospital, LMU Munich; Comprehensive Pneumology Center Munich (CPC-M), Member of the German Center for Lung Research (DZL), Munich, Germany; Institute of Epidemiology, Helmholtz Zentrum München, Neuherberg, Germany; Institut für Medizinische Informatik Biometrie Epidemiologie, Ludwig Maximilians University, Munich, Germany; Institut für Epidemiologie, Deutsches Forschungszentrum für Gesundheit und Umwelt, Neuherberg, Germany; Institute of Environmental Medicine, Karolinska Institutet, Stockholm, Sweden; Epidemiology Unit, Fondazione IRCCS Ca’ Granda Ospedale Maggiore Policlinico, Milan, Italy; Unit of Cancer Epidemiology, Department of Medical Sciences, University of Turin, Turin, Italy; Laboratory of Public Health and Population Studies, Department of Molecular Medicine, University of Padova, Padova, Italy; Epidemiology Unit, Istituto Dermopatico dell’Immacolata (IDI-IRCCS), Rome, Italy; Centre Armand-Frappier Santé Biotechnologie, Institut national de la recherche scientifique, Université du Québec, Laval, Québec, Canada; Dalla Lana School of Public Health, University of Toronto, Toronto, Canada; Cancer Care Ontario, Occupational Cancer Research Centre, Toronto, Canada; Division of Cancer Epidemiology and Genetics, National Cancer Institute, National Institutes of Health, Bethesda, MD, USA; Health Research Institute of the Principality of Asturias (ISPA), University of Oviedo and Ciber de Epidemiologia, CIBERESP, Oviedo, Spain; Department of Cancer Epidemiology and Prevention, N.N. Blokhin National Research Centre of Oncology, Moscow, Russia; Department of Environmental Epidemiology, Nofer Institute of Occupational Medicine, Lodz, Poland; National Public Health Centre, Budapest, Hungary; M Sklodowska-Curie National Research Institute of Oncology, Warsaw, Poland; Regional Authority of Public Health, Preventive Occupational Medicine, Banska Bystrica, Slovakia; Catholic University, Faculty of Health, Ružomberok, Slovakia; Roy Castle Lung Cancer Research Programme, University of Liverpool, Department of Molecular and Clinical Cancer Medicine, Liverpool, UK; National Institute of Public Health, Bucharest, Romania; Charles University, 1st Faculty of Medicine, Institute of Hygiene and Epidemiology & General University Hospital, Prague, Czech Republic; Masaryk Memorial Cancer Institute and Medical Faculty of Masaryk University, Dept. of Cancer Epidemiology & Genetics, Brno, Czech Republic; Faculty of Health Sciences, Palacky University, Olomouc, Czech Republic; Environmental Epidemiology Division, Institute for Risk Assessment Sciences, Utrecht University, Utrecht, The Netherlands; Stony Brook Cancer Center, Stony Brook University, Stony Brook, New York, USA; Department of Medical and Surgical Sciences, University of Bologna, Bologna, Italy

**Keywords:** Key terms job index, psychosocial, smoking, social prestige, tumor subtype

## Abstract

**Objectives:**

We investigated general job demands as a risk factor for lung cancer as well as their role in the association between occupational prestige and lung cancer.

**Methods:**

In 13 case–control studies on lung cancer, as part of the international SYNERGY project, we applied indices for physical (PHI) and psychosocial (PSI) job demands – each with four categories (high to low). We estimated odds ratios (OR) and 95% confidence intervals (CI) for lung cancer by unconditional logistic regression, separately for men and women and adjusted for study centre, age, smoking behavior, and former employment in occupations with potential exposure to carcinogens. Further, we investigated, whether higher risks among men with low occupational prestige (Treiman’s Standard International Occupational Prestige Scale) were affected by adjustment for the job indices.

**Results:**

In 30 355 men and 7371 women, we found increased risks (OR) for lung cancer with high relative to low job demands in both men [PHI 1.74 (95% CI 1.56–1.93), PSI 1.33 (95% CI 1.17–1.51)] and women [PHI 1.62 (95% CI 1.24–2.11), PSI 1.31 (95% CI 1.09–1.56)]. OR for lung cancer among men with low occupational prestige were slightly reduced when adjusting for PHI [low versus high prestige OR from 1.44 (95% CI 1.32–1.58) to 1.30 (95% CI 1.17–1.45)], but not PSI.

**Conclusions:**

Higher physical job demands were associated with increased risks of lung cancer, while associations for higher psychosocial demands were less strong. In contrast to physical demands, psychosocial demands did not contribute to clarify the association of occupational prestige and lung cancer.

Lung cancer risks are largely attributed to tobacco smoking, and occupational exposures to lung carcinogens (1, 2). Occupational social prestige and socioeconomic status are also identified as important risk factors, but – apart from supposed residual effects of smoking and exposure to occupational carcinogens – the pathways from occupational social determinants to lung cancer remain uncertain (3–6). Occupational conditions including psychosocial strain have been associated with elevated lung cancer risk (7) and may help to understand increased risks for occupations with a lower societal standing. Occupational prestige assigns a position in a perceived, hierarchical order of occupations that particularly captures work- and rank-related psychosocial demands. In addition, as an occupational indicator, it reflects material aspects of subject’s socioeconomic position (via income) and is directly linked with health outcomes by physical occupational hazards (8).

We extended analyses of the association between occupational prestige and lung cancer, previously identified in the international SYNERGY project (3), to investigate the role of further occupational exposures in this association. To cover a broad range of exposures and with regard to available job histories in SYNERGY, we applied two job-title based indices for general occupational demands (9) that have not yet been applied in the context of lung cancer. One was an index for environmental/physical demands, potentially also indicating effects of occupational carcinogens, and the other an index for psychosocial occupational demands. To our knowledge, to date, psychosocial demands have not been analyzed together with occupational prestige and lung cancer.

Before extending analysis of occupational prestige, we examined if the two occupational indices themselves were associated with lung cancer and thus appropriate for further analysis. This could additionally show if the job-title based indices are suitable for facilitated assessment of work environment risks when detailed occupational exposure information is not available.

Thus, in the first step, we analyzed the association of the two indices for general job demands and lung cancer and, in the second step, the role of these demands in the association of occupational prestige and lung cancer.

## Methods

The detailed methodology employed in SYNERGY has been published elsewhere (10). For this analysis of lung cancer and job indices, we included 13 European and Canadian case-control studies with 19 study centres of the SYNERGY dataset. Details and distribution of cases and controls are included in the supplementary material (www.sjweh.fi/article/3967), table S1. After exclusion of subjects with largely (>50%) missing or invalid occupational histories (N=1236) and missing smoking information (N=25), the dataset included 37 726 men and women (16 909 cases, 20 817 controls). To extend the previous social prestige analysis (3), we adapted inclusion criteria so that prestige analyses were restricted to 12 studies (18 study centres) and male gender (11 420 cases, 14 130 controls).

Job demands were assigned by two indices for general job demands (9). These indices were constructed and validated using German survey data for men and women and contain two/three dimensions of occupational demands: (i) a physical index (PHI) for ergonomic demands and environmental exposures (including acid, dust, fumes, climatic conditions, radiation, environmental tobacco smoke (ETS), dirt, noise, vibrations, low/glaring light, or need for protective clothing) and (ii) a psychosocial index (PSI) for mental (eg, overload, disruptions, low error tolerance), social (eg, lacking work control, conflicts, lacking support), and temporal (eg, on-call service, excessive working hours, shift work) demands. Originally, both indices may be summarized to an overall index, which we did not apply due to its high correlation with the PHI (Spearman correlation coefficient 0.95). We assigned both indices (range of 1–10 from low to high demands) to the subjects’ entire occupational histories and calculated time-weighted average (TWA) scores. TWA-scores were categorized into four categories: low (1, 2), lower middle (3–5), upper middle (6–8), and high (9, 10) demands (9). In sensitivity analyses, we recalculated scores disregarding the last ten years before diagnosis/interview to consider cancer latency. In the opposite direction, we used the last job to rather consider job demand effects on tumor promotion or progression.

To estimate lung cancer risks for job-demand indices (PHI, PSI), we calculated odds ratios (OR) with 95% confidence intervals (CI) by unconditional multiple logistic regression in a pooled analysis of all studies. We first adjusted for age (ln(age)) and study centre, then added smoking habits (smoking status [never (<1 pack-year in lifetime), former, current (including quitting smoking before <2 years), and other type of tobacco, including subdivision of former smokers by time since quitting smoking (2–7, 8–15, 16–25, >25 years)] and cigarette pack-years [ln(pack-years + 1)], and finally added ever employment in occupations and industries known to be associated with lung cancer with potential exposure to carcinogens (‘list A’ occupations) (12, 13) (final model). OR were estimated separately for main histological lung cancer subtypes [squamous cell carcinoma (SQCC), small cell lung cancer (SCLC), adenocarcinoma (ADC)]. In addition, job-demand indices were included as continuous variables to test for linear trends. To consider effects of individual studies, we compared results from the pooled analyses with meta-analyses (random-effects model) using the Paule–Mandel heterogeneity variance estimator (14) and displayed heterogeneity by I^2^.

For the prestige analysis, we adopted TWA prestige scores of Treiman’s Standard International Occupational Prestige Scale (SIOPS) (15), based on subject’s occupational history, and categorized it into low, medium, and high TWA prestige (3). We repeated models according to the original publication, adjusting for factors mentioned above (final model), education (<6, 6–9, 10–13, >13 years), and additionally the respective job index.

All calculations were performed with SAS, version 9.4 (SAS Institute Inc, Cary, NC, USA).

## Results

Descriptive information on the study population is shown in table 1. Both indices revealed higher job demands for cases than controls, with less pronounced differences for women and psychosocial exposures. TWA prestige was lower among cases.

**Table 1:** Study population. [IQR=interquartile range].

	Men				Women			
	
	Cases (N=13 791)		Controls (N=16 564)		Cases (N=3118)		Controls (N=4253)	
			
	N (%)	Median (IQR)	N (%)	Median (IQR)	N (%)	Median (IQR)	N (%)	Median (IQR)
Age (years)		63 (56–69)		63 (56–69)		61 (53–69)		61 (52–69)
Smoking status								
Non-smoker	393 (2.9)		4489 (27.1)		877 (28.1)		2689 (63.2)	
Former smoker	4829 (35.0)		7052 (42.6)		591 (19.0)		737 (17.3)	
Current smoker	8423 (61.1)		4680 (28.3)		1650 (52.9)		826 (19.4)	
Other types of tobacco only	146 (1.1)		343 (2.1)		0 (0)		1 (0)	
Cigarette pack-years in former and current smokers		39 (27–54)		25 (12–40)		31 (20–45)		17 (8–30)
Subtype of lung cancer								
Squamous cell carcinoma	5904 (42.8)				627 (20.1)			
Small cell	2226 (16.1)				502 (16.1)			
Adenocarcinoma	3391 (24.6)				1354 (43.4)			
Other/mixed	1401 (15.9)				622 (20.0)			
Missing	80 (0.6)				13 (0.4)			
Ever worked in ’list A’ occupations/industries ^a^								
Yes	2038 (14.8)		1559 (9.4)		80 (2.6)		53 (1.3)	
No	11753 (85.2)		15005 (90.6)		3038 (97.4)		4200 (98.8)	
Physical job exposure								
Low	854 (6.2)		1743 (10.5)		212 (6.8)		332 (7.8)	
Lower middle	2727 (19.8)		4906 (29.6)		1214 (38.9)		1963 (46.2)	
Upper middle	4739 (34.4)		5187 (31.3)		1358 (43.6)		1611 (37.9)	
High	5471 (39.7)		4728 (28.5)		334 (10.7)		347 (8.2)	
Psychosocial job exposure								
Low	740 (5.4)		1398 (8.4)		483 (15.5)		695 (16.3)	
Lower middle	4356 (31.6)		5695 (34.4)		691 (22.2)		1020 (24.0)	
Upper middle	6934 (50.3)		7528 (45.5)		1220 (39.1)		1797 (42.3)	
High	1761 (12.8)		1943 (11.7)		724 (23.2)		741 (17.4)	
Occupational prestige ^b^								
High	2209 (19.3)		4586 (32.5)					
Medium	3975 (34.8)		4847 (34.3)					
Low	5236 (45.9)		4697 (33.2)					

a Occupations and industries known to be associated with lung cancer.

b Analysis restricted to men and with reduced data set (11 420 cases and 14 130 controls).

In regression analysis (table 2), we found a gradient of lung cancer risks for increasing PHI in men [high versus low OR 1.74 (95% CI 1.56–1.93) and women (OR 1.62 (95% CI 1.24–2.11)] in the final models. Estimates for highest versus lowest PSI were lower than for PHI in men [OR 1.33 (95% CI 1.17–1.51)] and women [OR 1.31 (95% CI 1.09–1.56)]. Despite consistently significant tests for trend, risks were elevated just for the highest psychosocial demands among women. Only among men, risks decreased particularly after adjustment for smoking, and less after adjustment for ‘list A’ industries/occupations. Increased risks for higher job demands were detected for SQCC and SCLC, but not for ADC. Estimates of the random-effects model were slightly reduced compared to those of the one-stage regression [high versus low PHI: men OR 1.61 (95% CI 1.30–1.99), women OR 1.53 (95% CI 1.14–2.06) PSI: men OR 1.29 (95% CI 1.11–1.50), women OR 1.23 (95% CI 0.89–1.69)]. Statistically significant heterogeneity between the studies was only found for PHI in men (I^2^=60%, P<0.001). Both sensitivity analyses, assuming 10-year lag time and restriction to the last job, showed slightly reduced estimates for men and women, except slightly elevated OR for PHI for the last job among women (supplementary table S2) .

**Table 2:** Associations between lung cancer and job-exposure indices. [ADC=adeno carcinoma; PHI=physical index; PSI=psychosocial index; SCLC=small cell lung cancer; SQCC=squamous cell carcinoma]

Lung cancer type Job index	Men					Women				
	
	Cases	Controls	OR (95% CI) ^a^	OR (95% CI) ^b^	OR (95% CI) ^c^	Cases	Controls	OR (95% CI) ^a^	OR (95% CI) ^b^	OR (95% CI) ^c^
All lung cancers										
PHI										
Low	854	1743	1.00 ^d^	1.00 ^d^	1.00 ^d^	212	332	1.00 ^d^	1.00 ^d^	1.00 ^d^
Lower middle	2727	4906	1.08 (0.98–1.19)	1.06 (0.95–1.18)	1.05 (0.95–1.17)	1214	1963	0.99 (0.81–1.19)	1.07 (0.86–1.33)	1.07 (0.86–1.33)
Upper middle	4739	5187	1.82 (1.66–1.99)	1.47 (1.32–1.63)	1.43 (1.29–1.59)	1358	1611	1.30 (1.08–1.58)	1.34 (1.08–1.66)	1.32 (1.06–1.64)
High	5471	4728	2.27 (2.07–2.49)	1.82 (1.64–2.02)	1.74 (1.56–1.93)	334	347	1.44 (1.14–1.82)	1.66 (1.27–2.16)	1.62 (1.24–2.11)
PSI										
Low	740	1398	1.00 ^d^	1.00 ^d^	1.00 ^d^	483	695	1.00 ^d^	1.00 ^e^	1.00 ^w^
Lower middle	4356	5695	1.47 (1.33–1.62)	1.29 (1.15–1.44)	1.25 (1.12–1.40)	691	1020	0.98 (0.84–1.15)	1.01 (0.85–1.20)	1.00 (0.84–1.19)
Upper middle	6934	7528	1.75 (1.59–1.93)	1.41 (1.26–1.57)	1.35 (1.21–1.51)	1220	1797	0.98 (0.86–1.13)	0.97 (0.83–1.14)	0.96 (0.82–1.12)
High	1761	1943	1.75 (1.56–1.95)	1.37 (1.21–1.56)	1.33 (1.17–1.51)	724	741	1.46 (1.24–1.71)	1.32 (1.10–1.58)	1.31 (1.09–1.56)
SQCC										
PHI										
Low	283	1743	1.00 ^d^	1.00 ^d^	1.00 ^d^	26	332	1.00 ^d^	1.00 ^d^	1.00 ^d^
Lower middle	1061	4906	1.21 (1.05–1.40)	1.18 (1.01–1.38)	1.18 (1.01–1.38)	227	1963	1.43 (0.93–2.20)	1.50 (0.93–2.40)	1.50 (0.93–2.40)
Upper middle	2063	5187	2.27 (1.98–2.61)	1.84 (1.58–2.13)	1.81 (1.55–2.10)	306	1611	2.08 (1.36–3.20)	2.26 (1.41–3.62)	2.26 (1.41–3.62)
High	2497	4728	2.99 (2.60–3.42)	2.38 (2.05–2.76)	2.31 (1.99–2.68)	68	347	1.93 (1.18–3.16)	2.47 (1.44–4.25)	2.47 (1.43–4.25)
PSI										
Low	295	1398	1.00 ^d^	1.00 ^d^	1.00 ^d^	81	695	1.00 ^d^	1.00 ^d^	1.00 ^d^
Lower middle	1813	5695	1.54 (1.34–1.77)	1.34 (1.15–1.56)	1.30 (1.12–1.52)	117	1020	0.91 (0.67–1.24)	1.03 (0.73–1.46)	1.03 (0.73–1.45)
Upper middle	3043	7528	1.89 (1.65–2.16)	1.50 (1.30–1.74)	1.45 (1.25–1.68)	272	1797	1.21 (0.92–1.58)	1.35 (0.99–1.83)	1.34 (0.99–1.82)
High	753	1943	1.85 (1.59–2.16)	1.41 (1.19–1.68)	1.38 (1.16–1.64)	157	741	1.64 (1.22–2.21)	1.59 (1.14–2.23)	1.59 (1.14–2.22)
SCLC										
PHI										
Low	131	1743	1.00 ^d^	1.00 ^e^	1.00 ^d^	39	332	1.00 ^d^	1.00 ^d^	1.00 ^d^
Lower middle	406	4906	1.05 (0.85–1.29)	1.01 (0.81–1.26)	1.00 (0.81–1.25)	161	1963	0.76 (0.52–1.11)	0.77 (0.49–1.19)	0.76 (0.49–1.18)
Upper middle	791	5187	1.99 (1.64–2.42)	1.52 (1.23–1.87)	1.49 (1.21–1.83)	238	1611	1.29 (0.89–1.88)	1.31 (0.85–2.01)	1.29 (0.84–1.98)
High	898	4728	2.47 (2.04–3.00)	1.90 (1.55–2.34)	1.83 (1.48–2.25)	64	347	1.70 (1.09–2.65)	2.05 (1.21–3.48)	1.97 (1.16–3.34)
PSI										
Low	95	1398	1.00 ^d^	1.00 ^d^	1.00 ^e^	65	695	1.00 ^d^	1.00 ^d^	1.00 ^d^
Lower middle	720	5695	1.81 (1.45–2.26)	1.56 (1.23–1.98)	1.51 (1.19–1.92)	109	1020	1.15 (0.83–1.60)	1.19 (0.82–1.74)	1.18 (0.81–1.72)
Upper middle	1126	7528	2.17 (1.74–2.70)	1.66 (1.31–2.09)	1.59 (1.26–2.01)	197	1797	1.18 (0.87–1.59)	1.27 (0.90–1.80)	1.24 (0.88–1.76)
High	285	1943	2.21 (1.73–2.82)	1.65 (1.27–2.15)	1.61 (1.24–2.10)	131	741	2.10 (1.52–2.90)	1.98 (1.36–2.88)	1.93 (1.32–2.82)
ADC										
PHI										
Low	259	1743	1.00 ^d^	1.00 ^d^	1.00 ^d^	86	332	1.00 ^e^	1.00 ^e^	1.00 ^e^
Lower middle	841	4906	1.11 (0.96–1.30)	1.11 (0.95–1.31)	1.10 (0.94–1.30)	575	1963	1.06 (0.81–1.37)	1.10 (0.84–1.45)	1.10 (0.83–1.45)
Upper middle	1095	5187	1.41 (1.22–1.64)	1.17 (1.00–1.38)	1.14 (0.97–1.33)	551	1611	1.24 (0.95–1.61)	1.25 (0.95–1.65)	1.24 (0.94–1.64)
High	1196	4728	1.65 (1.42–1.91)	1.37 (1.17–1.60)	1.28 (1.09–1.51)	142	347	1.45 (1.05–1.99)	1.60 (1.15–2.24)	1.57 (1.12–2.20)
PSI										
Low	232	1398	1.00 ^d^	1.00	1.00	239	695	1.00	1.00	1.00
Lower middle	1105	5695	1.21 (1.03–1.41)	1.07 (0.91–1.27)	1.04 (0.88–1.23)	306	1020	0.92 (0.75–1.12)	0.92 (0.74–1.13)	0.91 (0.74–1.12)
Upper middle	1625	7528	1.34 (1.15–1.57)	1.11 (0.94–1.30)	1.06 (0.90–1.25)	523	1797	0.87 (0.73–1.04)	0.87 (0.72–1.05)	0.86 (0.71–1.04)
High	429	1943	1.35 (1.13–1.61)	1.10 (0.91–1.32)	1.07 (0.88–1.29)	286	741	1.17 (0.95–1.44)	1.09 (0.87–1.35)	1.08 (0.87–1.34)

a Adjusted for ln(age) and study centre.

b Adjusted for ln(age), study centre, smoking status including time since quitting (non-smoker, quitted 2-7, 8-15, 16-25, >26 years before interview/diagnosis, current smoker, other types of tobacco only) and cigarette pack-years (ln(pack-years+1)).

c Adjusted for ln(age), study centre, smoking status including time since quitting (non-smoker, quitted 2-7, 8-15, 16-25, >26 years before interview/diagnosis, current smoker, other types of tobacco only) and cigarette pack-years (ln(pack-years+1)) and ever employment in occupations and industries with potential exposure to carcinogens.

d P for linear trend <0.001.

e P for linear trend <0.05.

In the analysis of occupational prestige in men, lung cancer risks for low and medium versus high prestige [OR 1.44 (95% CI 1.32–1.58) and 1.23 (95% CI 1.13–1.34), respectively) were reduced by additional adjustment for PHI (low prestige 1.30 (95% CI 1.17–1.45), medium prestige 1.14 (95% CI 1.04–1.26)], but not for PSI [low prestige 1.46 (95% CI 1.33–1.61), medium prestige 1.24 (95% CI 1.14–1.35)].

## Discussion

In our analysis of lung cancer and job-demand indices in men and women, we found elevated lung cancer risks in particular for high physical job demands and less strong associations for psychosocial job demands. Adjustment for PHI reduced lung cancer risks of men with low occupational prestige but adjustment for PSI did not influence results.

We made use of the large SYNERGY database with its detailed smoking information and occupational histories. Previous SYNERGY analyses have identified possible residual effects of smoking due to potential information bias, lacking data on ETS, and possibly the inclusion of occasional smokers among non-smokers (defined by <1 cigarette pack-year) (3, 4). Similarly, we confirmed higher risks for higher job demands in the subtypes of lung cancer that are particularly related to smoking (SQCC, SCLC) and decreased risks for ADC (3, 4, 16). A potential limitation lies in the German database of the job indices, which we applied to international data. However, these data were all from (post-)industrial countries (Europe and Canada), and results of the random-effects model, considering study-specific variances, were similar to pooled estimates.

The applied job indices were constructed to allow assignment of general occupational demands on the basis of occupational job codes in the absence of more detailed information (9), which are included in SYNERGY for selected occupational carcinogens. We considered occupational lung carcinogens in general by ever exposure in ‘list A’ industries and occupations, a simplified exposure assessment. Occupational carcinogens therefore may also mainly account for the elevated risks for higher physical job index, ie, manual jobs, which may also include exposure to occupational fumes, dusts, and ETS. The reduction of risks of lower prestige occupations by adjustment for PHI might account for these previously uncaptured exposures to occupational carcinogens. Therefore, the physical index appears as crude but easily applicable proxy for occupational lung cancer hazards when only job titles were solicited.

Associations with lung cancer were lower for psychosocial compared to physical job demands. However, the PSI includes indicators for potential (lung) cancer risk factors, in particular chronic stress. Our results were similar to one study on lung cancer and work-related stress among men (7), while other studies did not find significantly increased risks (17, 18). We found an overall pattern of higher lung cancer risks for men, increasing with job demands, but no increase of risks for women with moderate psychosocial demands. The reasons for this finding remain unclear also because the job indices were constructed for men and women.

Generally, methodological issues in the assignment of job demands are critical in occupational cancer risk estimation as shown for two analyses of oesophageal cancer and psychosocial exposures (19, 20): one of which used personal questionnaires on job strain exposure and did not find an association for higher job strain (19), whereas in contrast, increased risks were detected when deducing job strain from job titles (20). However, in comparison to physical demands, derivation of psychosocial dimensions by objective job titles may be limited and dependent more on individual characteristics (9). This could explain why the observed associations were lower compared to the physical demands. This limitation has to be considered particularly for our analysis of occupational prestige and lung cancer, ie, we could have missed possible effects by adjusting for psychosocial job demands due to insufficient capture of these demands by job titles.

### Concluding remarks

The job-title-based indices suggested a role of occupational demands for lung cancer, beyond exposure to known occupational carcinogens, and their application in understanding work environment risks in the absence of detailed quantitative occupational exposure information. Lung cancer risks were particularly increased for higher physical job demands, likely due to capturing undetermined effects of occupational lung carcinogens. The index for psychosocial demands was less clearly associated with lung cancer, and – in contrast to physical demands – did not contribute to clarify the association of occupational prestige and lung cancer.

## Supplementary material

Supplementary material
