# Supplementary material for: Application of two job indices for general occupational demands in a pooled analysis of case–control studies on lung cancer
Source: Scand J Work Environ Health. 2021 Aug 31;47(6):475–81. doi: 10.5271/sjweh.3967 (PMC8504542; doi:10.5271/sjweh.3967)
Supplement: Supplementary material [file SJWEH-47-475-S001.pdf]

Application of two job indices for general occupational demands in a pooled analysis of case–control studies on lung cancer[1]

by Jan Hovanec, PhD,[2] Jack Siemiatycki, PhD, David I Conway, PhD, Ann Olsson, PhD, Pascal Guenel, MD, PhD, Danièle Luce, PhD, Karl-Heinz Jöckel, PhD, Hermann Pohlabeln, PhD, Wolfgang Ahrens, PhD, Stefan Karrasch, MD, Heinz-Erich Wichmann, PhD, Per Gustavsson, MD, PhD, Dario Consonni, MD, PhD, Franco Merletti, MD, PhD, Lorenzo Richiardi, MD, PhD, Lorenzo Simonato, MD, Cristina Fortes, PhD, Marie-Élise Parent, PhD, John R McLaughlin, PhD, Paul Demers, PhD, Maria Teresa Landi, MD, PhD, Neil Caporaso, MD, Guillermo Fernández-Tardón, PhD, David Zaridze, MD, PhD, Beata Świątkowska, PhD, Tamas Pándics, MD, Jolanta Lissowska, PhD, Eleonora Fabianova, MD, PhD, John K Field, PhD, Dana Mates, MD, Vladimir Bencko, MD, PhD, Lenka Foretova, MD, PhD, Vladimir Janout, PhD, Hans Kromhout, PhD, Roel Vermeulen, PhD, Paolo Boffetta, MD, MPH, Kurt Straif, MD, PhD, Joachim Schüz, PhD, Swaantje Casjens, PhD, Beate Pesch, PhD, Thomas Brüning, MD, PhD, Thomas Behrens, MD, PhD

1. *Supplementary material*
2. *Correspondence to: Jan Hovanec, IPA, Bürkle-de-la-Camp-Platz 1, 44789 Bochum, Germany. [E-mail: hovanec@ipa-dguv.de]*

Table S1. Description of selected case-control studies of the SYNERGY data base

Study	Country	Recruitment period	Cases[a]				Controls				Type of control recruitment[c]	Type of interview
			Resp[b]	Men	Women	n	Resp[b]	Men	Women	n		
AUT	Germany	1990–1995	77%	2656	499	3155	41%	2699	524	3223	P	Face-to-face
CAPUA	Spain	2000–2009	91%	640	51	691	96%	587	62	649	H	Face-to-face
EAGLE	Italy	2002–2005	87%	1525	358	1883	72%	1600	456	2056	P	Face-to-face
HdA	Germany	1988–1993	69%	838	161	999	68%	835	158	993	P	Face-to-face
ICARE	France	2001–2006	80%	2209	572	2781	76%	2742	713	3455	P	Face-to-faced
INCO	Czech. Rep.	1999–2002	94%	235	67	302	80%	292	158	450	H	Face-to-face
INCO	Hungary	1998–2001	90%	308	78	386	100%	243	56	299	H	Face-to-face
INCO	Poland	1998–2002	88%	547	237	784	88%	567	259	826	H/P	Face-to-face
INCO	Romania	1998–2002	90%	140	37	177	99%	149	68	217	H	Face-to-face
INCO	Russia	1998–2001	96%	519	79	598	90%	503	77	580	H	Face-to-face
INCO	Slovakia	1998–2002	90%	285	57	342	84%	236	48	284	H	Face-to-face
INCO	UK	1998–2005	78%	281	150	431	84%	572	327	899	P	Face-to-face
LUCA	France	1989–1992	98%	296	0	296	98%	293	0	293	H	Face-to-face
LUCAS	Sweden	1985–1990	87%	1009	0	1009	85%	2285	0	2285	P	Mail, telephone[d]

MONTREAL	Canada	1996–2002	85%	710	403	1113	69%	891	541	1432	P	Face-to-face[d]
PARIS	France	1988–1992	95%	161	8	169	95%	215	11	226	H	Face-to-face
ROME	Italy	1993–1996	74%	291	35	326	63%	261	61	322	H	Face-to-face
TORONTO	Canada	1997–2002	62%	192	184	376	71%	355	484	839	H/P	Face-to-face
TURIN/VENETO	Italy	1990–1994	79%	949	142	1091	80%	1239	250	1489	P	Face-to-face
Total		1985–2009		13,791	3118	16,909		16,564	4253	20,817		

[a] Histologically confirmed lung cancer cases
[b] Response rate
[c] H – hospital, P - population
[d] Including next-of-kin interviews

Table S2. Sensitivity analyses for associations between lung cancer and job-exposure indices: disregarding last 10 years of job history (lag time), restriction to last job

	Ten years lag time		Last job	
	Men	Women	Men	Women
Job index	OR (95% CI)[a]	OR (95% CI)[a]	OR (95% CI)[a]	OR (95% CI)[a]
PHI				
Low	1.00	1.00	1.00	1.00
Lower middle	1.02 (0.91-1.14)	0.99 (0.79-1.24)	1.02 (0.94-1.12)	1.30 (1.09-1.57)
Upper middle	1.41 (1.27-1.57)	1.23 (0.98-1.54)	1.34 (1.23-1.46)	1.57 (1.31-1.89)
High	1.66 (1.49-1.85)	1.48 (1.13-1.93)	1.62 (1.49-1.76)	1.75 (1.39-2.21)
PSI				
Low	1.00	1.00	1.00	1.00
Lower middle	1.23 (1.10-1.38)	0.95 (0.80-1.13)	1.26 (1.15-1.38)	1.05 (0.89-1.25)
Upper middle	1.32 (1.19-1.48)	0.99 (0.85-1.16)	1.30 (1.19-1.42)	0.92 (0.79-1.06)

High	1.32 (1.16-1.50)	1.25 (1.05-1.49)	1.28 (1.16-1.41)	1.29 (1.11-1.50)

PHI – physical index, PSI – psychosocial index
[a] Odds ratio with 95% confidence interval adjusted for ln(age), study centre, smoking status including time since quitting (non-smoker, quitted 2-7, 8-15, 16-25, >26 years before interview/diagnosis, current smoker, other types of tobacco only) and cigarette pack-years (ln(pack-years+1)) and ever employment in occupations and industries with potential exposure to carcinogens.
